# 

**Published:** 1906-07-07

**Authors:** 


### MEDICAL VACANCIES.

BIRMINGHAM, QUEEN'S HOSPITAL.—Honorary Surgeon.
BRIGHTON THROAT AND EAR HOSPITAL, Church Street, Queen's Road.—Non-resident House Surgeon.
CITY OF LONDON HOSPITAL FOR DISEASES OF THE CHEST, Victoria Park, E.—House Physician.
DERBYSHIRE ROYAL INFIRMARY.—Honorary Surgeon.
EGYPTIAN GOVERNMENT, MINISTRY OF EDUCATION.—Professor of Midwifery and Gynæcology. Also Medical Tutor and Registrar to Kasr-el-Ainy Hospital.
HOSPITAL FOR CONSUMPTION AND DISEASES OF THE CHEST, Brompton.—Resident House Physicians.
HOSPITAL FOR SICK CHILDREN, Great Ormond Street, London, W.C.—House Physician. Also Assistant Casualty Medical Officer. Also Radiographer.
HUDDERSFIELD INFIRMARY.—Junior House Surgeon.
HULL, ROYAL INFIRMARY.—Casualty House Surgeon.
LANCASTER, COUNTY LUNATIC ASYLUM.—Assistant Medical Officer.
LIVERPOOL STANLEY HOSPITAL.—Lady Honorary Medical Officer for Women's Diseases.
NEWCASTLE, CO. WICKLOW, ROYAL NATIONAL HOSPITAL FOR CONSUMPTION.—Medical Officer and Assistant.
NOTTINGHAM WORKHOUSE INFIRMARY.—Resident Medical Officer.
OXFORD, LITTLEMORE PAUPER LUNATIC ASYLUM.—Second Assistant Medical Officer.
PLYMOUTH, SOUTH DEVON AND EAST CORNWALL HOSPITAL.—Assistant House Surgeon.
ROYAL EAR HOSPITAL, Dean Street, Soho, London, W.—Clinical Assistants.
RYDE, ROYAL ISLE OF WIGHT COUNTY HOSPITAL.—Honorary Ophthalmic Surgeon.
STOCKPORT INFIRMARY.—Junior Assistant House Surgeon.
WEST HAM INFIRMARY, Whipps Cross Road, Leytonstone, N.E. Junior Assistant Medical Officer.
WOKINGHAM, BERKS, LONDON OPEN-AIR SANATORIUM, Pinewood.—Assistant Medical Officer.
WOLVERHAMPTON AND MIDLAND COUNTIES EYE INFIRMARY.—House Surgeon.

### NOTICE TO CORRESPONDENTS.

**All MSS., Letters, Books for Review, and other matters intended for the Editor should be addressed The Editor, "The Hospital," The Hospital Buildings, 28 & 29 Southampton Street, Strand, W.C.**

**The Editor cannot undertake to return rejected MS., even when accompanied by stamped directed envelope.**

### Notice to Advertisers and Subscribers.

**All Advertisements, Orders for Copies of The Hospital, and Business Communications should be addressed to THE MANAGER (Not to the Editor), The Hospital, 28 & 29 Southampton Street, Strand London, W.C.**

**The Cost of Subscription, post free, is as follows:—Per week, 3½d.; per quarter, 3s. 3d.; per half-year, 6s. 6d.; per annum, 13s. Foreign and Colonial:—Per annum, 19s. 6d., payable, in all cases, in advance.**

### Medical Appointments.

LIVERPOOL STANLEY HOSPITAL.

*Just Published.*

**An Important New Work for Students of Anatomy, Masseuses, and Nurses.**

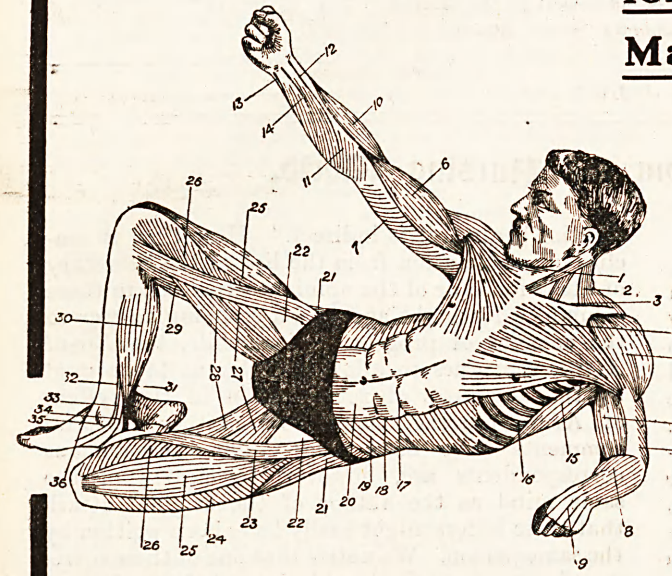

### MUSCLES AND NERVES

**3/6 net, 3/9 post free.**

**An Atlas of the Superficial Muscles and the Principal Motor Nerves of the Human Body for the use of Students of Anatomy and Nurses.**

**3/6 net, 3/9 post free.**

By LOUIS B. RAWLING, F.R.C.S.,

*Assistant Surgeon and Senior Demonstrator oj Anatomy, St. Bartholomew's Hospital.*

This Atlas contains a series of photographs of a well-developed male in such positions calculated to show off the superficial muscles to the best advantage. Opposite to each figure is a plan which not only illustrates the arrangement and direction of the muscles called into action, but also indicates the situation of the more important motor nerves.

The work cannot fail to be of the greatest practical utility to all Nurses and those who desire to gain a thorough insight into the superficial muscular formation of the human body, and obtain a general idea as to the position of the nerves which supply those muscles.

**The Scientific Press, Ltd.,**

*Catalogue of Nursing Manuals sent post free on application.*

**Publishers of Nursing Text-Books, Charts, and Case-Books of all Descriptions.**

**28 & 29 SOUTHAMPTON STREET, STRAND, LONDON, W.C.**

**Publications.**

### NEURASTHENIA, Clinical Lectures on.

By THOMAS D. SAVILL, M.D. Lond. 2nd Edition.

*Physician to the Hospital for Diseases of the Nervous System, &c. &c.*

"Lays down certain rules for treatment, and throws out hints of considerable practical value."—BRIT. MED. JOURN. "Should be in the hands of every practitioner."—TREATMENT.

H. J. GLAISHER, 57 WIGMORE STREET, W. **5s. net; 5s. 4d. by post.**

### TO BOOKBUYERS AND LIBRARIANS.

**W. H. SMITH & SON'S JULY CATALOGUE,** containing some 7,000 titles of Secondhand and New Remainder Books in all branches of literature, showing Reductions in Prices of 40% to 80%, is now ready, and will be sent post free upon application to W. H. SMITH & SON, 186 Strand, London, W.C.

Recently Published, Demy 8vo. cloth, 7s. 6d.

### THE INFLAMMATION IDEA IN GENERAL PATHOLOGY.

**By W. H. RANSOM M.D., F.R.S., &c.**

"The Essay has more than weight enough to recommend itself to the consideration of skilled inquirers specially interested in its problems, for it bears on every page evidences of original observation and independent thought."—*Scotsman.*

WILLIAMS & NORGATE, 14 Henrietta St., Covent Garden, London, W.C.

### CLEFT PALATE AND HARE-LIP:

**The Earlier Operation on the Palate.**

By **EDMUND OWEN,** M.B., F.R.C.S., Consulting Surgeon, St. Mary's Hospital, and to the Hospital for Sick Children, Great Ormond Street, London.

Pp. 111, with 32 Illustrations. Just Published. Price **2s. 6d.** net.

"A most valuable contribution to the literature of this subject."—*British Medical Journal.*

**London: BAILLIÈRE, TINDALL, & COX, 8 Henrietta Street, W.C.**

### WORKS BY DAVID WALSH, M.D.

**RÖNTGEN RAYS IN MEDICAL WORK.** *Third Edition.* 12s. 6d.

**THE HAIR AND ITS DISEASES.** 2s. 6d. net. A Standard Monograph. Baillère, Tindall, & Cox, London.

**GOLDEN RULES OF SKIN PRACTICE.** 1s. net. *Second Edition.* Wright & Co., Bristol.

**AGE AND OLD AGE: Handbook.** 2s 6d. R. A. Everett & Co., London.

JUST PUBLISHED. Price **2s. 6d.** net.

### CARCINOMA OF THE RECTUM.

**By F. SWINFORD EDWARDS, F.R.C.S.,**

Surgeon to the West London Hospital; Senior Surgeon to St. Peter's Hospital for Stone, and to St. Mark's Hospital for Fistula, &c.

London: BAILLIÈRE, TINDALL, & COX, 8 Henrietta Street, Covent Garden.

THE MOST EFFECTUAL BANDAGE FOR THE CURE OF ULCERS AND OTHER DISEASES OF THE LEG.

